# Sintering for High Power Optoelectronic Devices

**DOI:** 10.3390/mi16101164

**Published:** 2025-10-14

**Authors:** Hannes Schwan, Nihesh Mohan, Maximilian Schmid, Rocky Kumar Saha, Holger Klassen, Klaus Müller, Gordon Elger

**Affiliations:** 1 Institute for Innovative Mobility (IIMo), University of Applied Science Ingolstadt, Esplanade 10, 85049 Ingolstadt, Germany; nihesh.mohan@thi.de (N.M.); maximilian.schmid@thi.de (M.S.); rockykumar.saha@thi.de (R.K.S.); gordon.elger@thi.de (G.E.); 2 R&D Produkt Integration Backend, ams-OSRAM International GmbH, Leibnizstraße 4, 93055 Regensburg, Germany

**Keywords:** copper sintering, transient thermal analysis, optoelectronics

## Abstract

Residual-free eutectic Au80Sn20 soldering is still the dominant assembly technology for optoelectronic devices such as high-power lasers, LEDs, and photodiodes. Due to the high cost of gold, alternatives are desirable. This paper investigates the thermal performance of copper-based sintering for optoelectronic submodules on first and second level to obtain thermally efficient thin bondlines. Sintered interconnects obtained by a new particle-free copper ink, based on complexed copper salt, are compared with copper flake and silver nanoparticle sintered interconnects and benchmarked against AuSn solder interconnects. The copper ink is dispensed and predried at 130 °C to facilitate in situ generation of Cu nanoparticles by thermal decomposition of the metal salt before sintering. Submounts are then sintered at 275 °C for 15 min under nitrogen with 30 MPa pressure, forming uniform 2–5 µm copper layers achieving shear strengths above 31 MPa. Unpackaged LEDs are bonded on first level using the copper ink but applying only 10 MPa to avoid damaging the semiconductor dies. Thermal performance is evaluated via transient thermal analysis. Results show that copper ink interfaces approach the performance of thin AuSn joints and match silver interconnects at second level. However, at first level, AuSn and sintered interconnects of commercial silver and copper pastes remained superior due to the relative inhomogeneous thickness of the thin Cu copper layer after predrying, requiring higher bonding pressure to equalize surface inhomogeneities.

## 1. Introduction

**Figure 1:**
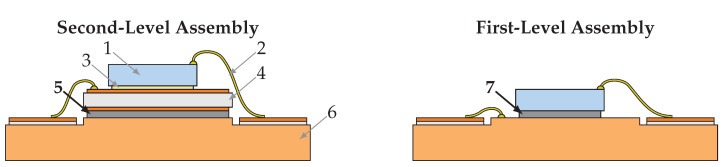
Second- and first-level packaging concepts: (1) optoelectronic bare die, (2) top contact wirebond (3) and (7) die-attach sinter interface (4) submount, e.g., ceramic carrier or copper–tungsten submount, (5) submount sinter interface (6) package platform, e.g., copper board, TO-can, copper micro channel cooler. In the case of Cu sintering, all metalization could be Cu.

Residual-free eutectic Au80Sn20 soldering was introduced about 30 years ago [1,2] and continues to dominate interconnection technology for high-power lasers and photodiodes. The residual-free process is particularly important to prevent contamination of optical surfaces, i.e., for p-side down mounted lasers [2]. AuSn provides a reliable non-corrosive solder joint and is also commonly used for high-brightness and high-power LEDs [3]. The residual-free approach was later adapted to SnAgCu solder bumps, preforms, and pastes [4]. Another key advantage of eutectic AuSn is its high melting point of 280 °C, which ensures stability during subsequent SnAgCu soldering for second-level LED package assembly. Since the late 1980s, sintering has evolved into a widely adopted die-attach method in power electronics [5,6]. In optoelectronics, initial approaches have applied Ag-based sintering to bare LED chip assembly [7,8] and laser modules [9,10], promising improved thermal performance over conventional AuSn solder and conductive adhesive interconnects. Remaining challenges include binder-derived residues—similar to flux soldering [11]—that may contaminate optical facets, as well as longer process times (3–5 min at 10–30 MPa [12] or 30–60 min without pressure [13]). Thus, at present, only wafer-, tile- or panel-level sintering processes can compete with fast AuSn soldering, where process speeds down to one second per die can be achieved. This paper proposes a packaging concept, depicted in Figure 1, that applies sintering on both the first and second level to achieve optoelectronic sub-assemblies with improved thermal performance. Based on their thermal bulk conductivity of around 200–400 W/mK—approximately four-eight times higher than that of conventional solders (~50 W/mK)—high-quality sinter interfaces offer significant performance potential. However, sintered interconnects are typically realized with bondline thicknesses of 20–40 µm, whereas AuSn interfaces produced industrially by sputtering or evaporation followed by thermode bonding can be up to an order of magnitude thinner [2]. As a result, much of the conductivity advantage is offset. Hence, only thin well-sintered interfaces can achieve superior thermal performances for optoelectronic sub-assemblies. From a materials perspective, AuSn offers low creep and very high shear strength, making it well suited for assembling components with significant thermomechanical mismatch, provided the brittle semiconductor can withstand the thermo-mechanical stresses [14]. In many cases, however, this is not achievable, and on the first level the substrate’s coefficient of thermal expansion must be matched to that of the die [9]. The creep behavior of Ag-sintered interconnects has been widely investigated [15]. For copper sintering, data remain limited, though thermo-mechanical shock tests suggest promising reliability for ceramic–copper pairings [16].

In comparison to commercial AuSn solder, a copper micro flake-based paste for pressure sintering, a silver nanoparticle paste for pressureless applications, and a new approach using a metal salt-based ink are investigated. In general, sintering processes are not inherently residual-free. It depends on the organic binders used in the inks/pastes and their role during sintering—whether they must remain after predrying to provide tackiness, reduce oxides, or protect particles from oxidation. Organic solvents are known to evaporate residual-free; for example, glycerin mixed with isopropanol is commonly used as a tacking agent. If the binder relied solely on such organics, the contamination risk would be comparable to soldering, as is the case for the new metal ink investigated here. To outperform a thin AuSn interconnect of about 2–4 µm bondline thickness, thin layers of sinterable copper ink are dispensed on the submounts, yielding comparable layer thicknesses. The metal salt is already decomposed to Cu nanoparticles during predrying to reduce the risk of contamination in the subsequent sintering process [17]. The evaporation of the complex binder is achieved to a large extent during the predrying process. This paper focuses on the transient thermal analysis (TTA) and investigates the thermal performance of the sintered interfaces compared to AuSn. The quality of the interconnects is assessed using scanning acoustic microscopy (SAM), cross-sectioning, and shear testing.

## 2. Materials and Methods

### 2.1. Sample Preparation

#### 2.1.1. Materials

Eutectic Au80Sn20 (80 wt.% Au and 20 wt.% Sn) solder preforms with a thickness of 30 µm were selected as the reference interconnect material for Ag and Cu sintering. The Ag nanoparticles (NP) paste (PL-LT-3002L) was obtained from DOWA Electronics Materials Co., Ltd. (JP). The particle-free Cu ink was prepared in-house using Cu(II) formate tetrahydrate (Cuf)-(98%) (Thermo Fischer Scientific, USA) and amino-2-propanol (A2P)-(93%) (Sigma-Aldrich, USA). In addition, a research Cu flake-based paste published in [18] was used to benchmark the performance of Cu interconnects realized with the developed Cu particle-free ink.

#### 2.1.2. Synthesis and Characterization of Cu Particle Free Inks

The particle-free Cu ink is prepared by mixing Cuf and A2P in a 1:2 molar ratio. The mixture is then agitated for 15 min at 1000 rpm in a planetary rotary paste mixer (Thinky SR500, JP). After mixing, the rheological behavior of the ink was analyzed using a viscometer (Thermo Scientific HaakeTM ViscotesterTM, USA). The prepared ink had a viscosity of 600–650 mPa·s. The thermal decomposition of the ink was analyzed using differential scanning calorimetry (DSC821 Mettler Toledo, USA) and thermal gravimetric analysis with mass spectroscopy (MS) (Mettler Toledo TGA/DSC 3+, USA). The samples were measured under a N_2_ atmosphere (flow rate: 30 L/min) with a heating rate of 10 °C/min up to 250 °C followed by an isothermal holding at 250 °C for 5 min. The particle morphology after decomposition was evaluated using a scanning electron microscope (Zeiss Auriga 40 Crossbeam FIB/SEM, GER). The in situ formed Cu nanoparticles layer after thermal decomposition of Cuf-A2P complex was evaluated for surface roughness and thickness using an optical light profilometer (Nanofocus µsurf, GER) prior to bonding.

#### 2.1.3. Sample Assembly

**Figure 2:**
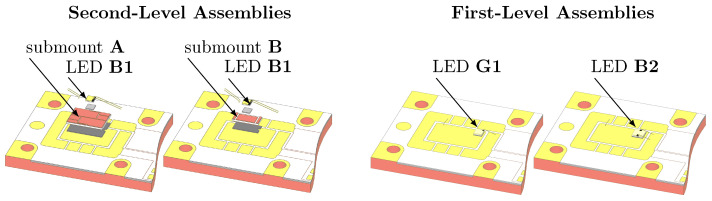
Exploded view of second- and first-level sample assemblies. The second-level assemblies were built up with a blue LED B1 (1 mm^2^) and two different sized ceramic submounts A (4.5 mm × 3.3 mm) and B (3.3 mm × 1.5 mm). On the first level, two different interconnect sizes were realized using LED G1 a green LED (1 mm^2^) and a blue LED B2 (2 mm^2^).

There are two different sets of sample assemblies prepared, one for investigation of the first-level interconnect with a directly sintered die on a copper baseplate (Ni-Pd-Au metalization) and another to adress the second-level interconnect with a ceramic submount sintered on the baseplate and subsequently a die soldered on the submount. As Figure 2 shows, for each set there are two variants taking different sinter interconnect sizes into account. Each set is prepared with different sinter materials, namely silver nanoparticle paste and particle-free Cu ink. In case of the first-level assemblies, additional samples were prepared with a Cu flake-based paste to realize Cu interconnects with low sinter pressure application.

**Figure 3:**
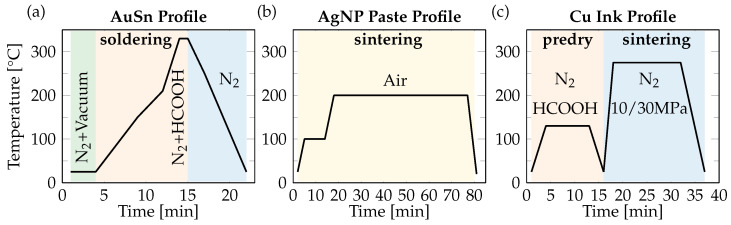
(**a**) Soldering profile of AuSn, (**b**) pressureless sintering profile of Dowa Ag nanoparticle paste, (**c**) pressure sintering profile of Cu particle free ink.

For reference, all samples were also realized with AuSn 30 µm preforms. The profile used can be seen in Figure 3a. Soldering was carried out in a batch reflow oven under formic acid-enriched N_2_ at 330 °C, i.e., above the eutectic temperature of AuSn, for 1 min. No pressure was applied to produce a thick AuSn interface, while applying pressure yielded a thin interface by squeezing out the solder. Although the soldering process applied in this paper is comparably long, it could also be performed as a rapid thermode bonding step under pressure on automated speed-optimized die bonders with cycle times as short as one second.

The Ag NP paste stored at −40 °C was kept at room temperature for one hour before printing. A 75 µm and a 150 µm stencil were used to print the Ag NP paste using a semi-automatic stencil printer (Go3v, PBT Works, CZE) with a motorized double squeegee (speed: 13 mm/s, squeegee pressure: 20 N) onto the baseplate. After printing, the submounts were placed on the wet paste using a pick and placer (FINEPLACER pico, GER). Predrying was performed at 100 °C for 10 min before sintering at 200 °C for 60 min (Figure 3b). For this, a two-step pressure-less sintering process was performed under air in a reflow oven (RSS 160-S, UniTemp, GER).

The Cu particle free ink is dispensed onto the substrate using the Musashi Image Master 350PC-Smart. The ink is then predried under a formic acid-enriched nitrogen atmosphere at 130 °C for 10 min in the reflow oven (RSS 160-S, UniTemp). The submounts are placed on the predried ink using the FINEPLACER pico. Pressure sintering is performed by applying 30 MPa (Second level: submounts) and 10 MPa (First level: LEDs) bonding pressure at 275 °C for 15 min under a constant flow of nitrogen (5 L/min) in an open bonding chamber (FINEPLACER sigma, GER), as shown in Figure 3c.

For the transient thermal analysis, i.e., thermal performance characterization, of the assembled structures, dies comprising high-power vertical-thin-film (VTF) GaN LEDs with varying footprints were selected. As shown in Figure 2, for the second-level assemblies, a blue bare die LED with an area of 1 mm^2^ and die bond bar pad, referred to as B1, was soldered onto the submounts and wire-bonded using a total of four 30 µm gold wires, including two bonds for the anode contact. To realize different interconnect sizes on the second level, two direct bond copper (DBC) ceramic submounts of different dimensions but identical layer thicknesses (Cu: 75 µm, AlN: 380 µm, Cu: 75 µm) and metalization (NiPdAu) were chosen. The larger submount A measured 4.5 mm × 3.3 mm, compared to the smaller submount B at 3.3 mm × 1.5 mm. The first-level assemblies were realized, with both a 1 mm^2^ green VTF corner-pad LED, designated G1, and a 2 mm^2^ blue dual-pad LED B2 to achieve different interconnect sizes on the first level.

### 2.2. Analysis Methods

#### 2.2.1. Interconnect Quality Analysis Methods

The assembled samples are non-destructively analyzed using scanning acoustic microscopy (SAM, Sonoscan, Nordson Test & Inspection, USA) to inspect defects (such as voids and delaminations) in the interconnect. The first-level samples are measured from the baseplate side using a 100 MHz transducer to observe the interconnect quality unobstructed by the LED. Similarly, a 50 MHz transducer is used to resolve the quality of the interconnection between the baseplate and the submounts. In addition, the samples are subjected to destructive shear testing (XYZTEC Condor Sigma Lite Shear tester, NLD) with a shear height of 25 µm and a shear rate of 200 µm/s for LEDs and 500 µm/s for submounts to evaluate the shear strength of the respective interconnects. The cross sections of the interconnect material (AuSn solder, Ag NP paste and Cu ink) are prepared by metallographic grinding and fine polishing. They are then evaluated using scanning electron microscopy (Zeiss Auriga 40 Crossbeam FIB/SEM, GER).

#### 2.2.2. Transient Thermal Analysis

TTA is a non-destructive characterization method commonly used in power- and optoelectronics to evaluate the thermal performance of high-power semiconductor modules. In this study, the method is applied to investigate the interconnects of different materials by determining their thermal resistance $R_{\mathrm{th}}$. The thermal impedance $Z_{\mathrm{th}}\left(t\right)$, i.e., the time resolved $R_{\mathrm{th}}$, defines the transient thermal response of the semiconductors junction temperature $\Delta T_{j}\left(t\right)$ according to an applied heating power step $\Delta P_{h}$. In the case of an LED, $P_{h}$ is derived from the applied electrical power $P_{\mathrm{el}}$ adjusted by the wall-plug-efficiency η to account for the light emission.

(1) $$Z_{\mathrm{th}}\left(t\right)=\frac{\Delta T_{j}\left(t\right)}{\Delta P_{h}}=\frac{\Delta T_{j}\left(t\right)}{\Delta P_{\mathrm{el}}\;\cdot \;\left(1-\eta \right)}$$

**Figure 4:**
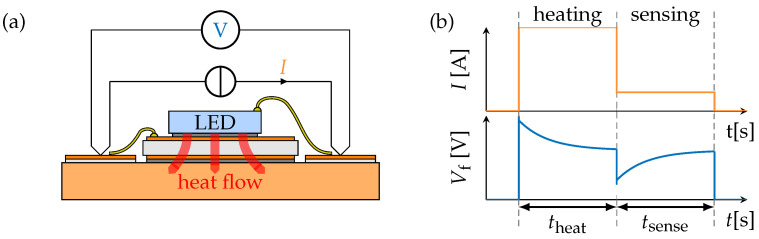
(**a**) Schematic of the TTA method for an LED module and (**b**) waveforms of heating current and measured forward voltage.

Low values of $Z_{\mathrm{th}}\left(t\right)$ are desired, as they indicate preferable thermal performance of the module and therefore correspond to reduced operating temperatures of the semiconductor. The TTA method resolves the layers along the heat flow path from the source of power dissipation (LED) to the baseplate (Figure 4a). Consequently, interconnects with reduced thermal conductivity or defects such as inhomogeneous interfaces, delaminations, cracks, and voids show a comparable increase in the $Z_{\mathrm{th}}\left(t\right)$ curve.

The TTA measurement procedure is defined for LEDs in [19] but is theoretically applicable for all types of semiconductors. As shown in Figure 4b, thermal excitation of the package is realized by a pulsed current $I_{\mathrm{heat}}$ through the semiconductor, leading to a power loss step and a heat flow toward the temperature-stabilized baseplate. The duration $t_{\mathrm{heat}}$ of the heating phase is determined for the sample to reach thermal equilibrium; subsequently, a small sense current is applied, which is expected to not contribute to further self-heating.

This enables measurement of the junction temperature $\Delta T_{j}\left(t\right)$ during cool down in $t_{\mathrm{sense}}$ using a temperature-sensitive parameter (TSEP), such as the forward voltage $V_{f}\left(t\right)$, which shows a linear dependence and typical sensitivity SEN in the range of −1 to −2 mV/K:

(2) $$\Delta T_{j}\left(t\right)=\frac{\Delta V_{f}\left(t\right)}{SEN}$$

The measured temperature change together with the applied heating power defines the time-resolved thermal impedance, representing the thermal influence of the different materials according to their thermal properties and position in the layer stack. Thinner layers with low thermal capacity and resistance close to the junction affect the earlier time domain, whereas layers closer to the heat sink are represented towards the end. However, prior to TTA measurements, both factors η and SEN must be individually determined for each sample. An alternative approach to assess the thermal performance of TTA-measured modules is the normalized logarithmic time derivative B(z) of $Z_{\mathrm{th}}\left(t\right)$, which can be evaluated independently of $P_{H}$, i.e., η, and SEN of the semiconductor [20]:

(3) $$B\left(z\right)=log\left(\frac{dZ_{\mathrm{th}}\left(z\right)}{dz}\right)=log\left(\frac{d}{dz}V_{f}\left(z\right)\right)-log\left(SEN\;\cdot \;P_{\mathrm{el}}\;\cdot \;\left(1-\eta \right)\right).$$

Due to the exponential nature of $Z_{\mathrm{th}}\left(t\right)$, the information of the change in heat flow is more pronounced on the logarithmic time scale. Therefore, one first substitutes z=ln(t) before calculating the logarithmic time derivation, Equation (3).

**Figure 5:**
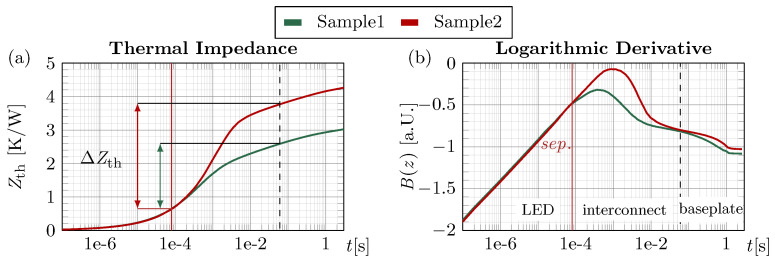
(**a**) $Z_{\mathrm{th}}\left(t\right)$ and (**b**) B(z) curves for two first-level assemblies with different die-attach materials.

Since SEN and $P_{h}$ act only as scaling factors, they appear as an offset in the logarithmic representation; thus, shifting different B(z)-curves onto each other in intervals of equal heat paths eliminates these dependencies. This is illustrated in Figure 5 for two first-level samples with different interconnects. The $Z_{\mathrm{th}}\left(t\right)$ curves (Figure 5a) separate around 80 µs due to an obstructed heat flow in the die attach of Sample 2, before following the same thermal path again from approximately 0.6 ms onward, corresponding to the baseplate time domain. Both points in time, indicated by vertical lines, are difficult to identify in the $Z_{\mathrm{th}}$-representation but become evident in the B(z)-curves. The change in thermal impedance within this interval, $\Delta Z_{\mathrm{th}}$, reflects a comparable increase mainly attributed to the die interconnect. However, since $Z_{\mathrm{th}}\left(t\right)$ represents the superposition of all thermal responses [21,22] in the thermal path, $\Delta Z_{\mathrm{th}}$ also includes contributions from other layers. Defining the end of influence of a specific layer in the time domain is challenging in both representations, which is why a shared reference point of equal heat flow, i.e. converging B(z) curves, in the late domain is used. Methods such as the structure function overcome this by separating layer contributions via deconvolution, though resolution and arithmetical precision are critical for resolving thin interconnects, increasing the computational effort. Overall, the $\Delta Z_{\mathrm{th}}$ method provides an effective way to compare the module thermal performance, but does not yield the exact $R_{\mathrm{th}}$ of an individual layer.

## 3. Results

### 3.1. Realization of Ultra-Thin Sinterable Cu Layer Using Particle Free Cu Ink

**Figure 6:**
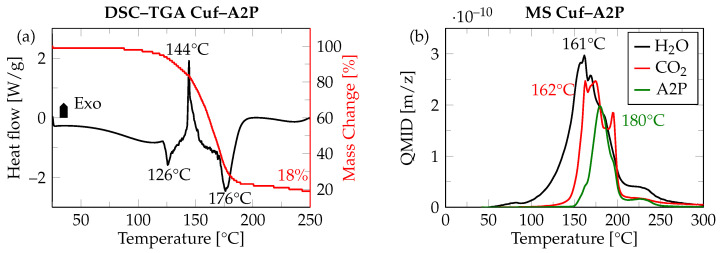
(**a**) DSC–TGA curve indicating change in heat flow (W/g) and mass change (%) during the thermal decomposition of Cuf–A2P complex [24]. (**b**) MS curve indicating the release of gaseous byproducts during the thermal decomposition of Cuf–A2P complex [24].

Cuf as the Cu metal precursor and A2P as the complexing ligand were selected for the preparation of Cu particle-free ink, based on previous investigations [23,24,25]. Figure 6a shows the DSC and TGA curve for the thermal decomposition process of the Cuf–A2P complex. The DSC curve shows an exothermic peak at 144 °C with onset at 126 °C, which is attributed to the decarboxylation reaction (indicated by the release of CO_2_ and H_2_O) and subsequent reduction of the Cu^2+^ ion to metallic Cu^0^. The narrow peak during this exothermic reaction also indicates the faster nucleation of in situ generated Cu nanoparticles, which is crucial for the realization of the ultra-thin sinterable Cu layer [23]. This is followed by an endothermic peak at 176 °C, which is attributed to the evaporation of A2P. The TGA data show the final mass loss at 18 wt. (%), which corresponds to the theoretical amount of Cu in the complex after all volatile components have left the system. This is further validated by the MS analysis, where the release of various gaseous byproducts such as CO_2_, H_2_O, and A2P are shown (see Figure 6b). This confirms that the Cuf–A2P complex decomposes completely into Cu metal and volatile byproducts (CO_2_, H_2_O and A2P), leaving no solid residues behind, which is crucial for optoelectronic applications.

**Figure 7:**
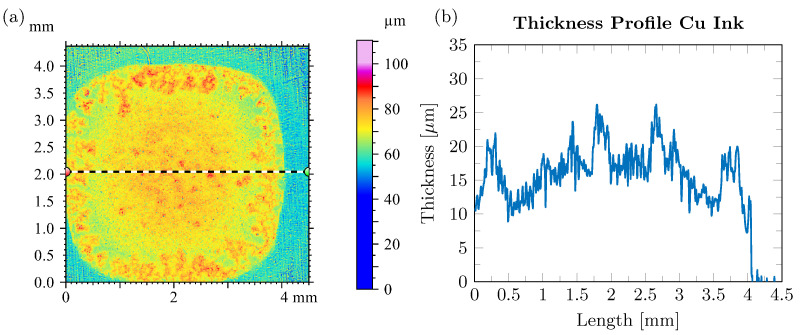
(**a**) Surface profile of the Cu ink after thermal decomposition (predrying) at 130 °C for 10 min. (**b**) Thickness profile of Cu ink after thermal decomposition (predrying) at 130 °C for 10 min.

In the two-step Cu ink sintering process (Figure 3c), the pre-drying step is performed first at 130 °C for 10 min under formic acid-enriched N_2_ (to prevent the oxidation of the nascent Cu nanoparticles generated in situ). This step corresponds to the onset of the decomposition of the Cuf–A2P complex as observed in the DSC, to allow the formation of a thin Cu layer and subsequent evaporation of A2P [24]. The isothermal holding time of 10 min at 130 °C further allows a slow and controlled evaporation of A2P. Figure 7a,b show the surface and thickness profile of Cu ink after the pre-drying step. It is evident from the surface profile that the formed Cu layer has surface inhomogeneities, resulting in a non-uniform thickness of 17 ± 5.7 µm. The TGA curve (Figure 6a) shows that the Cu ink has 18 wt. (%) Cu after thermal decomposition, indicating that there is a large volume shrinkage creating a network of loosely connected Cu nanoparticles, leading to a porous and rough morphology as observed in Figure 7b. Multiple approaches have been investigated to solve this problem. One common approach would be to modify the solvent evaporation rate using a slower heating ramp rate. Another approach would be to increase the wetting of the dispensed ink on the substrate by plasma pretreatment. However, the final high temperature sintering step is also crucial as it helps in densifying the predried Cu film and reducing the surface roughness by application of pressure.

### 3.2. Analysis of Soldered and Sintered Interconnects

First- and second-level interconnects are investigated with samples as illustrated in Section 2, Figure 2. The first-level assemblies comprised LEDs with die sizes of 1 mm^2^ and 2 mm^2^, designated as G1 and B2, respectively. These interconnects are prepared with AuSn preforms (thin: AuSn1, thick: AuSn2), Ag nanoparticle paste (thin: AgNP1, thick: AgNP2), Cu particle-free ink (CuInk), and a Cu flake-based paste (CuFL). The second-level assemblies with a 1 mm^2^ LED B1 on a larger submount A (4.5 × 3.3 mm^2^) and smaller submount B (3.3 × 1.5 mm^2^) were prepared and evaluated in a similar manner. All samples are based on a 15.5 × 27.0 mm^2^ Cu baseplate of 1.5 mm height and NiPdAu metalization.

**Figure 8:**
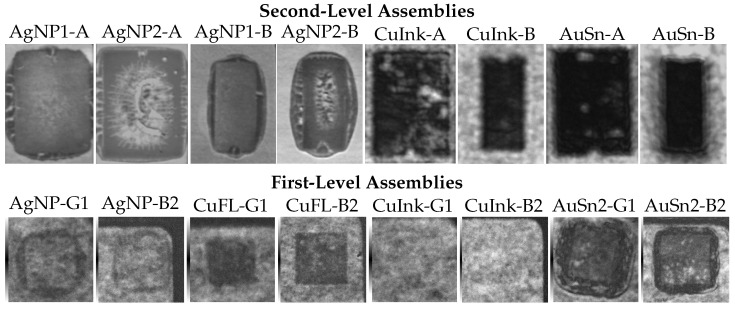
SAM images of AgNP, CuInk, and AuSn interconnect for second-level samples with submounts A and B and first-level interconnects AgNP, CuFL, CuInk, and AuSn for LED G1 and B2.

Figure 8 presents SAM images taken from the baseplate side, focusing on the first- and second-level interconnects of different sizes. At the second level, the thin AgNP1 submount A and B interconnects, stencil-printed with 75 µm thickness, exhibit a uniform interface. The pressureless sintering process thus produced a homogeneous void-free interconnect. In contrast, the thicker AgNP2 interconnects, printed with a 150 µm stencil, show a comparatively poor interface for both submount sizes. The bright regions in the SAM images (Figure 8: AgNP2-A, AgNP2-B) at the center of the interconnects correspond to voids, attributed to stronger degassing of the organics. For the smaller submount B, the reduced area and paste volume appear to reduce this effect. At the first level, however, AgNP was dot-dispensed and compressed during LED placement to form thin interfaces comparable to AuSn1, also resulting in degassing issues.

The CuInk samples were pressure-sintered at 30 MPa for the second-level modules, whereas the LEDs G1 and B2 limited the sinter pressure to only 10 MPa for the first-level assemblies. This difference is reflected in the resulting interconnects: the interfaces of submounts A and B (CuInk-A, CuInk-B) appear dense and, aside from minor irregularities, relatively uniform, while not resolvable at the first level, indicating a very poor connection to the LED as suggested by the surface roughness measurements (Figure 7) and later confirmed by cross-sectional analysis. For this reason, additional first-level Cu interconnects using a flake-based paste (CuFL) were realized, which, according to the SAM analysis, exhibit a dense interface with a relatively low void rate, comparable to the reference samples soldered pressure-less with AuSn preforms. The interfaces of the AuSn-soldered reference samples display some voids at the first level (AuSn2-G1, AuSn2-B2) and, for the larger submount A, also at the second level, attributed to flux degassing. In contrast, the smaller submount B samples appear unaffected and show a uniformly soldered interconnect throughout.

**Figure 9:**
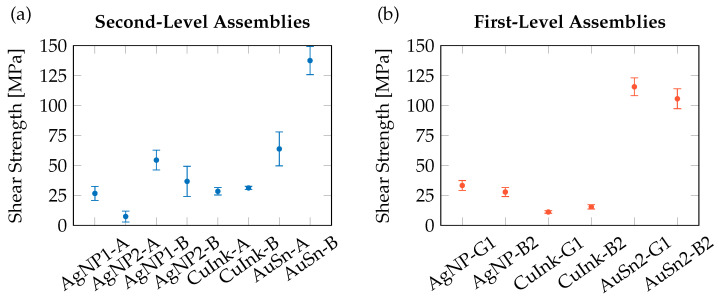
Shear strength of AgNP, CuInk, and AuSn interconnect for (**a**) second-level samples with submounts A and B and (**b**) first-level interconnects AgNP, CuInk, and AuSn for LED G1 and B2.

**Figure 10:**
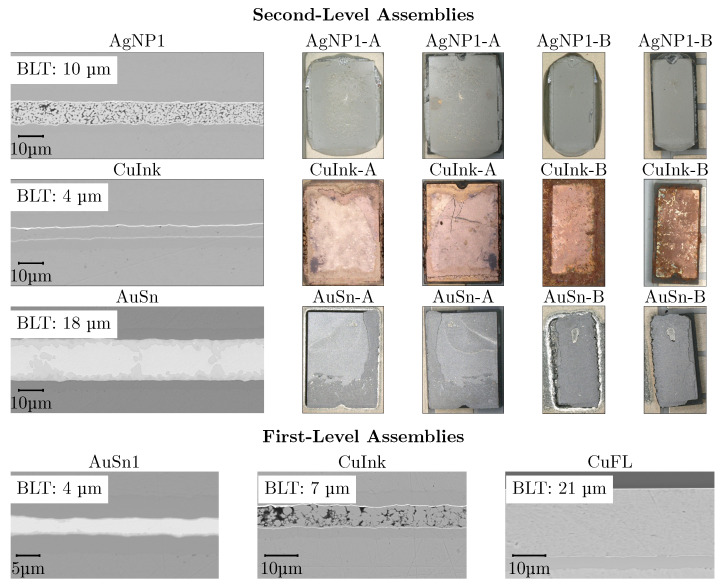
SEM cross section of the first- and second-level interconnects with corresponding shear modes (left images: baseplate) and (right images: submount).

The shear test results are summarized in Figure 9, with corresponding shear modes shown in Figure 10. At the second-level, AuSn interfaces exhibited shear strengths of 138 ± 12 MPa (submount B, small) and 64 ± 14 MPa (submount A, large), representing a 58–60 % increase over thin AgNP1 (B: 55 ± 8 MPa, A: 27 ± 6 MPa). Cu ink samples (B: 31 ± 1 MPa, A: 28 ± 3 MPa) performed comparably to AgNP, with AuSn yielding a 56–78 % higher strength. Notably, the larger AuSn submounts A showed a ~46 % drop in shear strength compared to samples of submount B, attributed to impaired degassing and, hence, void formation, visible in the SAM images (Figure 8). For the die attach (Figure 9b), AuSn soldering again achieved the highest shear strengths (116 ± 7 MPa and 105 ± 8 MPa depending on die size), about 72–76 % higher than AgNP (G1: 33 ± 4 MPa, B2: 28 ± 4 MPa), consistent with the irregular interfaces of the SAM images (Figure 8). Compared with Cu ink (G1: 11 ± 1 MPa, B2: 15 ± 2 MPa), AuSn showed an improvement of 87–91 %, in agreement with the unresolved SAM images, indicating that the applied sinter pressure of 10 MPa was insufficient to compensate for surface roughness after predrying (Figure 7b).

Figure 10 shows the shear mode in AgNP1-A and AgNP1-B after shear test (left: baseplate and right: submount). The sheared surfaces of both appear to have the interconnect material, suggesting the failure occurred within the interconnect layer itself. For AgNP1-A, the sheared surface shows roughness suggesting the presence of voiding even for the thin variant. However for AgNP1-B, the surface roughness is lower with a more homogenous bond, indicating less voiding and resulting in higher shear values with a cohesive shear mode in concurrence with SAM images (Figure 8). Both the Cu Ink samples (CuInk-A, CuInk-B) show a mixed shear mode in the interconnect. The darker areas indicate that the interconnect material itself failed, while the brighter areas suggest some delamination at the interface. However, the thin BLT (~4 µm) and reduced degassing of both samples result in uniform shear values with fewer deviations (see Figure 9), providing a homogeneous Cu interconnect in concurrence with the SAM images (Figure 8). The shear mode for AuSn1 appears mostly cohesive with partly delaminated structures at the adhesion interface of the substrate and the submounts. The high shear strength in AuSn1 is due to the fine grained AuSn phase and crack inhibition effect of the adjacent Au_5_Sn interlocked phase [26]. Additionally, the delamination appears more pronounced in case of larger submount A compared to the smaller submount B, which is evident from the void formation seen in Figure 8, resulting in a significant drop in the shear strength (Figure 9).

Figure 10 also compares the bondline thicknesses (BLT) of the first- and second-level assemblies. For the second-level AgNP1 assemblies, the BLT was about 10 µm with a porous structure caused by voids from organic degassing during pressureless sintering. The CuInk assemblies showed a very thin BLT of 4 µm; this dense homogeneous layer results from applying 30 MPa, preventing pores and inclusions. AuSn assemblies exhibit a thicker BLT of 18 µm, yet remain uniform due to the pressureless soldering of preforms. To benchmark against typical AuSn die attaches (2–4 µm), first-level AuSn1 assemblies were prepared with a BLT of 4 µm using pressure. Since the LEDs could only withstand 10 MPa, CuInk assemblies were processed under the same conditions, yielding a 7 µm layer height. Cross sections reveal a porous non-uniform structure with poor shear strength (as seen in Figure 9). In order to benchmark Cu interconnects, Cu flake paste samples were stencil-printed, producing a 21 µm BLT with a thick, yet homogeneous, bondline.

### 3.3. Transient Thermal Analysis Results

The TTA measurements are performed with a heating current of $I_{\mathrm{heat}}$ = 1 A to reduce the influence of the rather long wirebonds (~3 mm). The sense current $I_{\mathrm{sense}}$ was set to 20 mA, and equal heating and sensing times of $t_{\mathrm{heat}}$ = $t_{\mathrm{sense}}$ = 3 s were used to reach thermal equilibrium. The sensitivity and efficiency of the different LEDs were determined in advance, the latter with a spectroradiometer (Gigahertz-Optik BTS2048-VL-TEC, GER). Due to noise caused by switching from the heating to the sensing current, extrapolation was performed until $t_{\mathrm{cut}}$ = 35 µs (fit window: 35–80 µs) according to [19]. The offset compensation in the B(z) representations was done with a shifting interval of 20–80 µs, in the time domain of the Si carrier layer, assuming equal heat flow in the LEDs.

**Figure 11:**
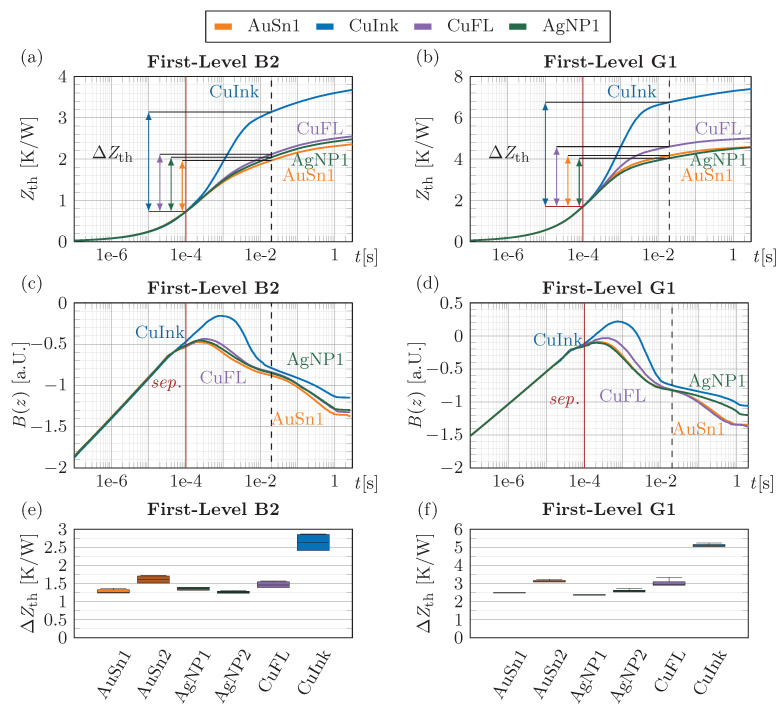
Thermal impedance $Z_{\mathrm{th}}\left(t\right)$ of the (**a**) directly bonded 2 mm^2^ LED B2 and (**b**) 1 mm^2^ LED G1, logarithmic time derivative B(z) of $Z_{\mathrm{th}}\left(t\right)$ for (**c**) the B2 and (**d**) G1 LED samples. Comparison of the thermal resistances $\Delta Z_{\mathrm{th}}$ of thin AuSn1, thick AuSn2, thin AgNP1, thick AgNP2, CuFL flake paste and CuInk for the first-level interconnected (**e**) B2 LEDs and (**f**) G1 LEDs.

The results of the TTA measurements on the first-level interconnect samples, i.e., the directly bonded LEDs, are shown in Figure 11. The thermal impedance $Z_{\mathrm{th}}\left(t\right)$ of the respective best sample of each sample group with size 3 is depicted in Figure 11a for the 2 mm^2^ LED B2 and in Figure 11b for the 1 mm^2^ LED G1. In addition, the related B(z) curves are shown in Figure 11c and Figure 11d, respectively. Until approximately 100 µs, the curves are overlapping, i.e., the thermal impedance is only influenced by the properties of the LEDs. From this point onwards, the curves start to separate according to the quality and thermal properties of the different interconnect materials. The separation becomes more pronounced in the B(z) curves and is indicated with a red vertical line. The influence of the interconnect ends when the B(z) curves converge again at 0.2 ms. However, the change in the thermal impedance $\Delta Z_{\mathrm{th}}$ within this interval is solely for comperative purposes and should not be converted to a thermal conductivity, as the recombination (dashed line) occurs, due to different heat spreading, in the time domain of the baseplate. The $\Delta Z_{\mathrm{th}}$ values for assessment of the thermal performance are depicted in Figure 11e,f for both interconnect sizes of B2 and G1. It should be noted that even for the small 1 mm^2^ area, the thermal interface caused by the bulk properties of the interconnect material would only be 0.07 K/W (AuSn, thermal conductivity λ = 58 W/mK) for a 4 µm BLT and 0.02 K/W assuming copper or silver sintered material with λ = 200 W/mK. Those small thermal resistances can hardly be separated. Therefore, the contribution of the interfaces are measured relatively, comparing the different interconnects between each other. It can be observed that a thinner AuSn interface (AuSn1) with approximately 4 µm BLT shows a smaller thermal impedance change (B2: 1.29 K/W, G1: 2.52 K/W) than AuSn2 with 18 µm (B2: 1.64 K/W, G1: 3.14 K/W) as expected. Note, the values do not scale with the thickness, due to the included interface and adjacent layer contributions. However, the heat flow in these layers seems to reflect the change of the interconnect size, as they almost perfectly scale with a factor of 2 comparing the two different LEDs B2 and G1. The AgNP paste does not show this expected thickness dependence, since the results of the thin AgNP1 (B2: 1.37 K/W, G1: 2.38 K/W) and thicker AgNP2 (B2: 1.26 K/W, G1: 2.59 K/W) samples only present an increase for the smaller LED G1. This can be attributed to a lower quality of thinner sinter interconnects by degassing, as indicated by the SAM images (Figure 8). However, the results of the silver sintered samples are very comparable to those of AuSn for both thickness variants. The thermal performance of the pressure-sintered copper flake paste CuFL (B2: 1.49 K/W, G1: 3.07 K/W) with a BLT of 21 µm is slightly better than the AuSn2 results with even an ~3 µm thinner interface, demonstrating the potential of Cu sintering. As already indicated by the SAM analysis and cross-sectioning of the CuInk samples on the first level, the thermal performance (B2: 2.7 K/W, G1: 5.12 K/W) is rather poor, which can be attributed to the non-sufficient sintering pressure of 10 MPa, limited by the LEDs.

Overall, the AgNP and CuFL sintered interfaces compare well in terms of thermal performance with AuSn die attachs, while the pressure sintering process for the CuInk needs further improvement.

**Figure 12:**
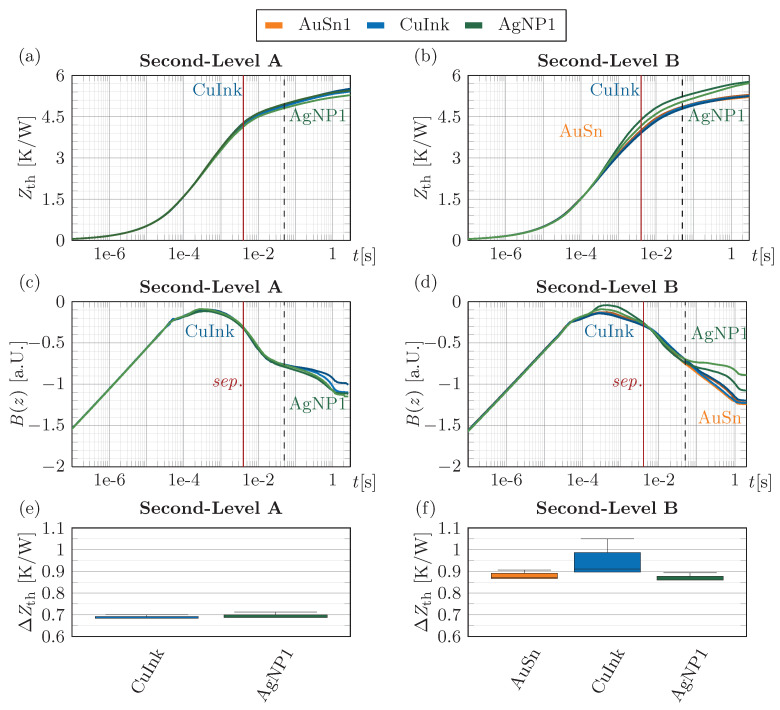
Thermal impedance $Z_{\mathrm{th}}\left(t\right)$ of the (**a**) second-level samples with large A and (**b**) small submount B, logarithmic time derivative B(z) of $Z_{\mathrm{th}}\left(t\right)$ for (**c**) larger A and (**d**) small submount samples B. Comparison of the thermal resistances $\Delta Z_{\mathrm{th}}$ of AuSn, AgNP and Cu for the second-level interconnect (**e**) large A and (**f**) small submount B.

The results of the second-level interconnects in Figure 12 show that the same CuInk can thermally perform on the same level as AuSn when sufficient pressure is applied during sintering. The relevant time domain was defined by curve separation due to the die attach at 4 ms, with an additional separation at 50 ms, corresponding to the baseplate and the thermal interface material to the temperature-controlled plate. The determined $\Delta Z_{\mathrm{th}}$ in this interval, shown in Figure 12e,f, therefore reflects the influence of the second-level interconnect from the ceramic submount to the baseplate. Both the thin AgNP1 (A: 0.7 K/W, B: 0.88 K/W) and the CuInk (A: 0.7 K/W, B: 0.96 K/W) provide comparable overall thermal performance to AuSn (B: 0.88 K/W), independent of submount size. However, the effect of bondline thickness differences (AgNP1: 10 µm, CuInk: 4 µm, AuSn: 18 µm) is difficult to resolve, since a 1 µm increase in AuSn BLT would only add ~0.004 K/W for submount B, assuming complete heat spreading. Between submounts B and A, a factor of 3 would be expected if the full area contributed to heat transfer, yet the results scale only by about 1.3. Thus, as discussed, calculation of the thermal conductivity of the interface material is not possible due to incomplete heat spreading and contributions from other layers. The thermal analysis proved that the CuInk is a viable alternative to commercial Ag based sinter pastes and AuSn solder for second-level interconnects, as very thin BLTs and comparably high conductivity values can be achieved, if a dense and homogeneous interface is realized.

## 4. Conclusions

Transient thermal analysis was utilized to benchmark thin ( ~4 µm) Cu particle-free ink interconnects against conventional AuSn soldering and Ag sintering for optoelectronic submodules. The copper ink was dispensed on a Cu baseplate and predried at 130 °C to facilitate in situ generation of Cu nanoparticles by thermal decomposition of the metal salt before sintering. Subsequently, both ceramic submounts holding high power bare die GaN LEDs and the LEDs themselves were pressure sintered onto the baseplates at 275 °C for 15 min.

The TTA results showed nearly identical thermal performance between AuSn and the Cu ink for the submount interconnects, with a deviation of only ~0.08 K/W. However, the thermal analysis also revealed that achieving reliable interfaces currently requires a high bonding pressure of 30 MPa, challenging for most dies. The pressure application was necessary to compensate for inhomogeneities in the predried Cu nanoparticle layer caused by the dispensing and drying process. To address this, future work will focus on optimizing ink viscosity for ink-jet printing to achieve a uniform particle layer, enabling lower bonding forces and shorter processing times. In addition, the predrying process will be optimized to obtain even smaller nanoparticles immersed in a small amount of organics which provides tacking of the dies and evaporates before sintering. As a competitive process with a superior thermal starting point, sintering on ceramic tile- or panel-level using existing equipment of facilities, i.e., sinter presses, is targeted.

## Data Availability

Data are contained within the article.
